# The mechanism of a green fluorescent protein proton shuttle unveiled in the time-resolved frequency domain by excited state *ab initio* dynamics†
†Electronic supplementary information (ESI) available. See DOI: 10.1039/c7sc02803b


**DOI:** 10.1039/c7sc02803b

**Published:** 2018-01-02

**Authors:** Greta Donati, Alessio Petrone, Pasquale Caruso, Nadia Rega

**Affiliations:** a Dipartimento di Scienze Chimiche , Università di Napoli ‘Federico II’ , Complesso Universitario di M.S.Angelo , via Cintia , I-80126 Napoli , Italy . Email: nadia.rega@unina.it ; Tel: +39 081 674207; b Italian Institute of Technology , IIT@CRIB Center for Advanced Biomaterials for Healthcare , Largo Barsanti e Matteucci , I-80125 Napoli , Italy

## Abstract

We simulated an excited state proton transfer in green fluorescent protein by excited state *ab initio* dynamics, and examined the reaction mechanism in both the time and the frequency domain through a multi resolution wavelet analysis. This original approach allowed us, for the first time, to directly compare the trends of photoactivated vibrations to femtosecond stimulated Raman spectroscopy results, and to give an unequivocal interpretation of the role played by low frequency modes in promoting the reaction. We could attribute the main driving force of the reaction to an important photoinduced softening of the ring–ring orientational motion of the chromophore, thus permitting the tightening of the hydrogen bond network and the opening of the reaction pathway. We also found that both the chromophore (in terms of its inter-ring dihedral angle and phenolic C–O and imidazolinone C–N bond distances) and its pocket (in terms of the inter-molecular oxygen’s dihedral angle of the chromophore pocket) relaxations are modulated by low frequency (about 120 cm^–1^) modes involving the oxygen atoms of the network. This is in agreement with the femtosecond Raman spectroscopy findings in the time-frequency domain. Moreover, the rate in proximity to the Franck Condon region involves a picosecond time scale, with a significant influence from fluctuations of nearby hydrogen bonded residues such as His148. This approach opens a new scenario with *ab initio* simulations as routinely used tools to understand photoreactivity and the results of advanced time resolved spectroscopy techniques.

## Abstract 2

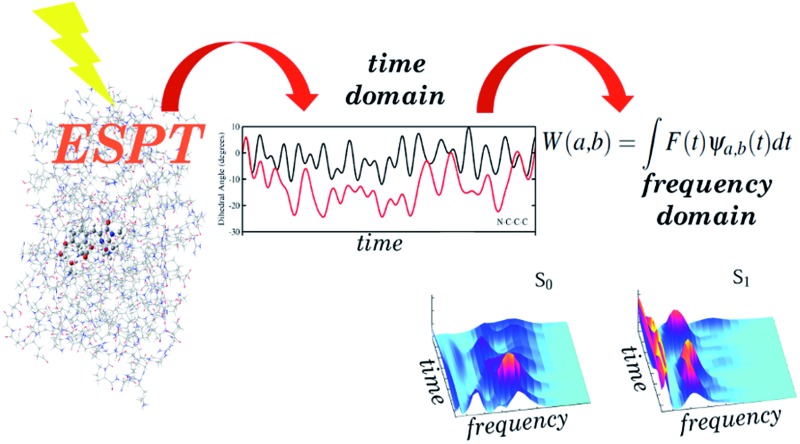
A new time-resolved vibrational analysis unveils the mechanism of an excited state proton shuttle in green fluorescent protein.

## 1 Introduction

Exploring far-from-equilibrium phenomena represents one of the main goals for modern chemistry. Photoreactivity, a time-resolved optical property of great importance in both biochemistry and material science, is now explored in very short time and high resolution frequency windows by advanced time-resolved spectroscopic techniques.^1^–^5^ These experiments deliver plenty of new information about electronic and nuclear photoinduced rearrangements after short times, which opens pathways for photophysical and photochemical events, although the comprehension of real-time molecular dynamics remains difficult. New tools for interpretation are therefore needed, and disciplines such as theoretical and computational chemistry are particularly challenged.

The case of the proton shuttle occurring in green fluorescent protein (GFP) is emblematic in this context. GFP, first isolated from the jellyfish *Aequorea Victoria* (wtGFP), is employed in many fields as a marker in cell biology^6^–^8^ and is responsible for an increasing interest in the whole class of fluorescent proteins that are widely investigated nowadays .^9^–^11^

The photoreactivity of GFP is due to its chromophore, *p*-hydroxybenzylideneimidazolinone (HBDI), which in the electronic ground state is present in both its neutral (A) and anionic (B) form, with a neutral form population six times higher than the anionic one under physiological conditions.^12^ After the excitation, an excited state proton transfer (ESPT) reaction takes place leading to the formation of an anionic form, denoted as I*, responsible for the emission at around 508 nm.^12^ The reaction consists of three proton transfers occurring along a hydrogen bond network linking the chromophore (the first proton donor), a crystallographic water molecule, the Ser205 residue and the Glu222 residue as the final acceptor^13^,^14^ and is characterized by biphasic kinetics with time scales of 3 and 10 ps.^12^,^14^–^16^

Although the photophysics and photoreactivity of GFP have been widely explored by computational studies,^17^–^29^ the driving force of the GFP ESPT reaction and its mechanism and kinetics are still the subject of a lively scientific debate.^30^–^32^ Mathies and coworkers, on the basis of femtosecond stimulated Raman spectroscopy (FSRS) results, pointed out the significant role of a photoexcited low frequency mode involving the chromophore in promoting the approach of the phenolic ring to the hydrogen bonded water molecule.^4^ In contrast, the importance of modes involving heavy atoms did not emerge in other studies.^26^,^33^

Focusing on theoretical studies, reaction energy profiles aimed to validate the ESPT mechanism have been obtained at several levels of theory, including higher order wavefunction based methods,^17^,^34^–^36^ or combining density functional theory (DFT) with quantum mechanical/molecular mechanics (QM/MM) approaches.^37^,^38^ *Ab initio* molecular dynamics (AIMD) studies have also been proposed on GFP models,^39^,^40^ and on the entire GFP at the QM/MM level of theory, also including quantum effects.^26^ In this latter study however, the modelling did not include the photoinduced structural relaxation and the process in the excited state remains endergonic. A recently calculated intrinsic reaction coordinate on a reduced GFP model did include the rearrangement of the chromophore pocket, although this provided the analysis of a limited region of the potential energy surface accessible to the system without fully accounting for the protein matrix effects.^41^

We propose here for the first time an innovative theoretical protocol to disentangle such a complex photoinduced reaction. This approach is general and can be easily employed for the investigation of both reaction events and other far-from-equilibrium processes directly in the frequency domain. Our computational strategy consists of two main steps: (1) an accurate simulation of the reaction by excited state *ab initio* molecular dynamics (AIMD)^42^,^43^ based on energy and energy derivatives calculated by time dependent density functional theory (TD-DFT)^44^–^47^ and (2) analysis of the trajectories based on the multi-resolution time resolved wavelet transform,^48^–^50^ employed for the first time as an analytical method to study photoreactivity. The wavelet protocol, successfully implemented by us to analyse excited state non-equilibrium solvation,^51^ allows the following of vibrational band evolutions and their couplings associated to significant molecular structural changes (*i.e.* during a photoreaction) or related to other perturbations affecting the molecule (microsolvation changes) in non-equilibrium conditions. The vibrational signals activated during a photoinduced reaction can be compared to the vibrational modes in the ground state at equilibrium and the modes promoting the reaction can be recognized. In this way a molecular picture of a photoactivated process can be achieved with *real time* resolution. This procedure is powerful when photoactivated modes of interest are collective and strongly coupled in nature, as in the case of the GFP ESPT. Instantaneous shifts and couplings of transient vibrational bands can be followed and analysed, providing a direct and fruitful comparison with experimental signals, such as those recorded by FSRS. As a matter of fact, we obtained an excellent agreement with the FSRS results from Mathies, reproducing the peculiar out of phase relaxation of some marker bands (C–O and C–N stretching modes of the chromophore). Moreover, we could undoubtedly attribute the photoinduced gain of conformational freedom of the chromophore to the activation of low frequency modes governing and augmenting the planarity of the hydrogen bond wire, thus facilitating the reaction.

## 2 Methods

### 2.1 GFP *ab initio* molecular dynamics

**Fig. 1:**
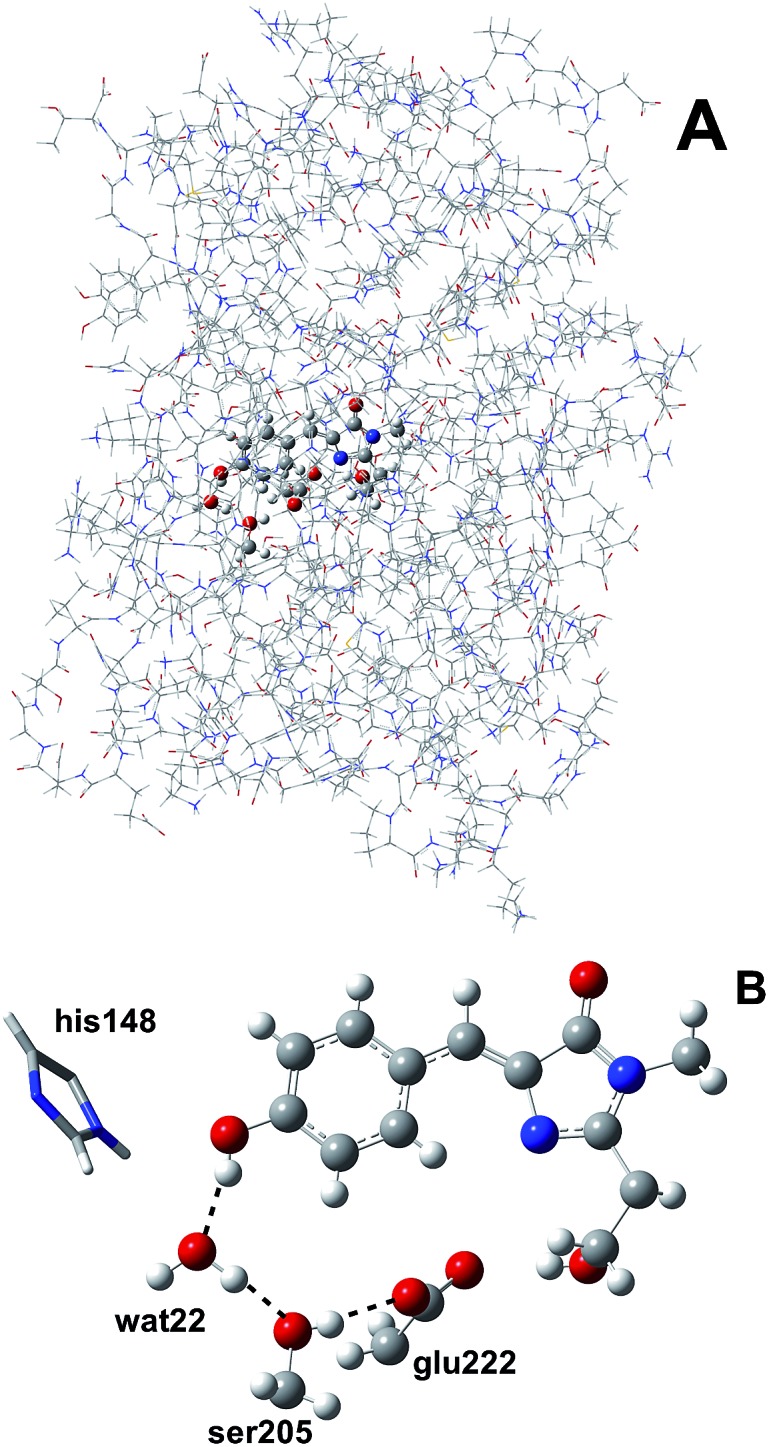
(A) The ONIOM partition of GFP adopted in this study. The ONIOM model (ball and stick representation) includes the chromophore completed by the Ser65 side chain, the hydrogen bonded crystallographic water molecule and the side chains of Ser205 and Glu222. (B) The residues and hydrogen bond network involved in the ESPT reaction in the ball-and-stick representation. The His148 residue, involved in a hydrogen bond with the chromophore phenolic ring, is also included. His148 is described at the MM theory level in this paper, so it is shown in the tube representation.

We simulated GFP by a quantum mechanics/molecular mechanics (QM/MM) description, according to the ONIOM partition scheme.^52^–^55^ The ESPT shuttle involves the phenolic oxygen of the chromophore (Otyr) as the first donor, the hydrogen bonded crystallographic water (wat), the Ser205 and finally the Glu222 residue as the final acceptor. Therefore the ONIOM model included the chromophore completed by the Ser65 side chain, the water molecule and the side chains of Ser205 and Glu222 (see Fig. 1).

DFT was chosen as the *high* level of theory and used in both the time independent and time dependent^44^–^47^ formalisms to define the ground and first singlet excited states potential energy surfaces (PESs), respectively.

This energy potential was used to calculate a minimum energy structure in the ground state, considered as the reference, and to run AIMD simulations within the Born-Oppenheimer approximation^42^,^43^ in both the S_0_ and the S_1_ states. As a matter of fact, our interest was to simulate the ultrafast photorelaxation of GFP, therefore we could safely rely on a single PES treatment. A more complete description of GFP photophysics would have required accounting for non-adiabatic processes,^56^ affecting the GFP dynamics at longer times. This, however, would have been computationally unfeasible for the ONIOM energy potential considered here.

Five AIMD trajectories were collected in the S_1_ state starting from structures and velocities extracted from the ground state sampling, which is representative of the Franck–Condon region of the PES. One of the starting structures was close to the minimum energy, at least with regards to the chromophore and the chromophore pocket arrangement; the remaining ones were chosen randomly.

In four S_1_ trajectories (TRJI-IV) an ESPT event was observed within one picosecond. In one case the run failed after a small number of steps, and this S_1_ trajectory is not discussed in the following sections.

Further computational details and test calculations performed to validate the level of theory that was adopted in the molecular dynamics simulations are reported in the computational methods section.

### 2.2 Vibrational and energetic analysis

The main goal of the present work was to analyze the ESPT mechanism in both the time and the frequency domain. The time-resolved vibrational analysis is based on a continuous wavelet transform,^50^,^57^,^58^ which simply replaces the sine and cosine waves used in the Fourier transform with wavelet ones:1
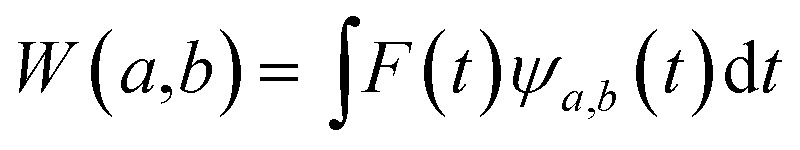
where *F*(*t*) is the signal in the time domain, in our case several structural parameters extracted from AIMD trajectories, and *Ψ*_*a*,*b*_(*t*) is the wavelet function. The wavelet functions are defined as dilated and translated versions of a mother wavelet and can be expressed in this way:2
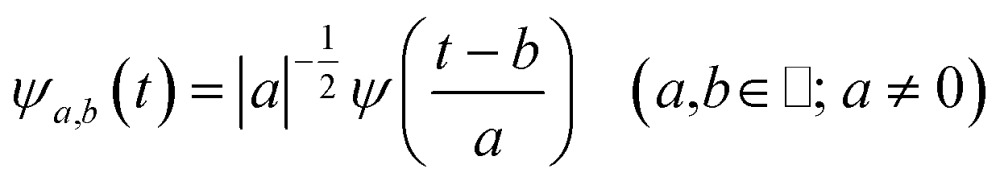
where both *a* and *b* are real numbers. The *a* parameter is the scale or dilation proportional to the inverse of the frequency, and *b* is the position or translation parameter.

The wavelet transform is a multiresolution analysis technique that can simultaneously capture contributions from different vibrational modes, providing the unique ability to unveil the presence of multiple frequency contributions in the analysed signal with optimized time-frequency resolutions of both the high and low frequency modes. This can be very convenient compared to standard fixed window analysis techniques (*i.e.* short-time Fourier transform), where once an analyzing width is chosen, a fixed time and frequency resolution is obtained. By changing the *a* parameter the wavelet function is stretched or compressed allowing an accurate analysis of both high and low frequency contributions, while the *b* parameter allows the wavelet function to shift along the signal, thus obtaining a temporal localization of the frequencies. Of course, the frequency resolution (Δ*v*) is inversely proportional to the time resolution (Δ*t*) for a given mode in the signal, obeying the Heisenberg uncertainty principle (Δ*v*Δ*t* ≥ 1/(4π)).^59^–^61^ On the basis of the smooth oscillating nature of our signals, we chose the Morlet function as the mother wavelet.^50^,^51^,^58^ A set of discretized scales, {*a*_*i*_}, was also used which allowed us to simultaneously optimize the time-frequency resolution. In particular adopting the procedure presented in ref. 50, resolutions of about 2–5, 10 and 20 cm^–1^ were obtained in the 10–1300, 1300–3000 and 3000–4000 cm^–1^ regions, respectively.

We calculated the wavelet power spectra by plotting the |*W*(*v*,*t*)|^2^, converting the scale to wavenumber.^62^ The value of the magnitude (|*W*(*v*,*t*)|^2^) represents the intensity of the instantaneous frequency contribution to the signal.

The intensity of the wavelet power spectra represents a time-resolved and out-of-equilibrium (for the excited state parameters) version of the spectroscopical analysis from the classical MD simulation, and not the quantum electric dipole cross-section in terms of normal modes.

In order to facilitate the assignment of vibrational bands, we performed harmonic frequency calculations on a GFP reduced model in the neutral form, representing the PT reactant. Harmonic frequencies were obtained in both the S_0_ and the S_1_ states (in the latter case by calculating TD-DFT energy second derivatives numerically) and considered as limit values to be compared to transient photoactivated vibrational bands. S_0_ and S_1_ minimum energy structures of the GFP model were optimized in a previous study.^41^

Finally, rigid scans of the ground state gas-phase minimum of the chromophore in its anionic form were also performed in both the ground and excited states by changing the chromophore N–C–C–C dihedral angle to values ranging from –30 and +30 degrees (see ESI† for more details). This analysis supports the results found from the dynamics that clearly reveal the crucial role of the chromophore conformation in ESPT mechanism.

## 3 Results

### 3.1 GFP AIMD in the ground state

**Fig. 2:**
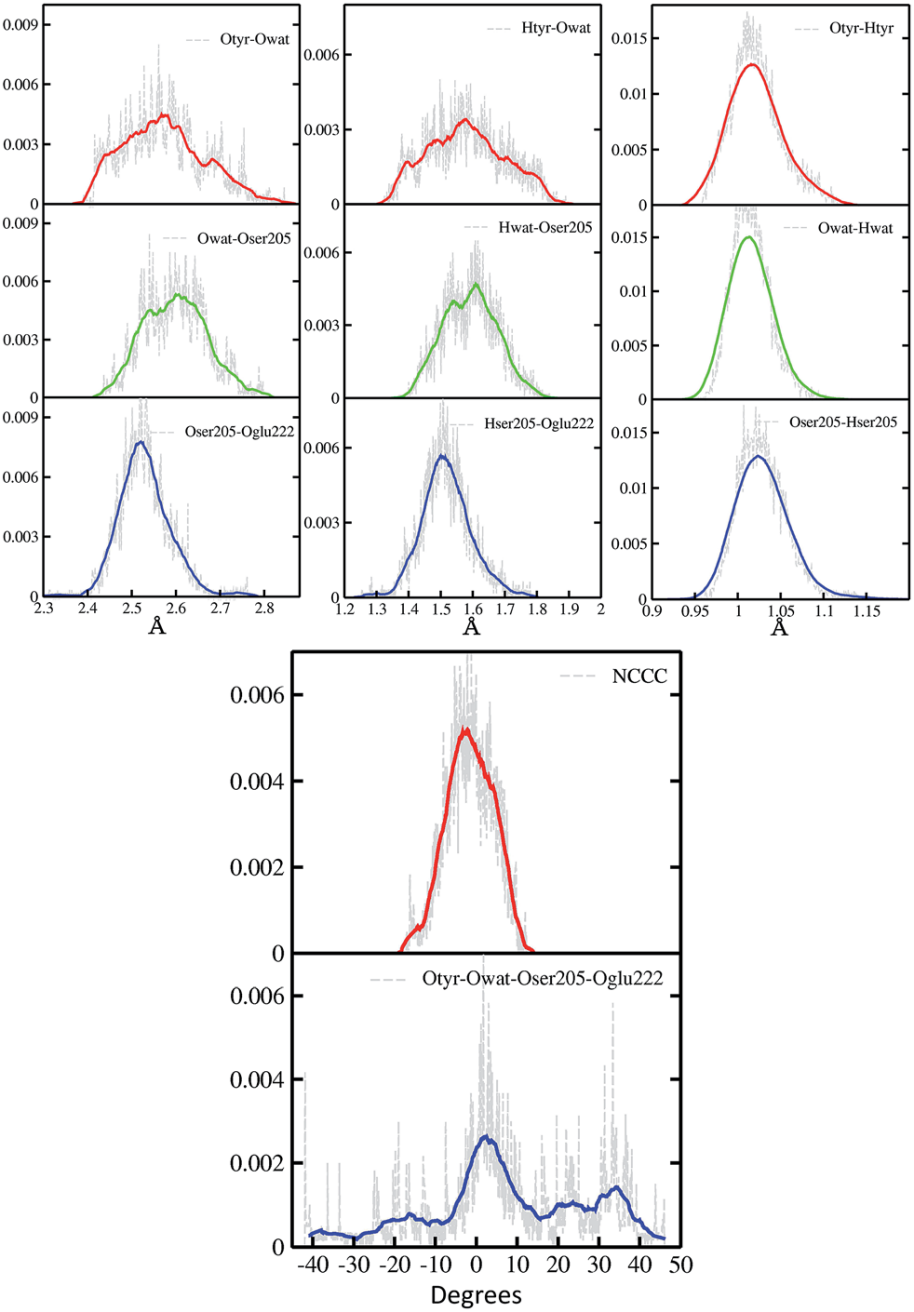
Time-averaged distributions of distances (Å) and dihedral angles (degree) of the GFP PT network obtained from the S_0_ AIMD simulation. Top left column: oxygen–oxygen distances; top middle column: hydrogen–acceptor oxygen distances; top right column: hydrogen–donor oxygen distances; bottom column: chromophore N–C–C–C and hydrogen bond network Otyr–Owat–Oser205–Oglu222 dihedral angles. Average values, shown by coloured curves, are calculated every 100 points for the Otyr–Owat–Oser205–Oglu222 dihedral angle, and every 50 points in all other cases.

In Fig. 2 a summary of the structural analysis of the GFP AIMD in the ground state is presented, including the time-averaged distribution of distances and dihedral angles of the GFP hydrogen bond network supporting the proton transfer.

Regarding the intramolecular hydrogen–donor oxygen distances (Otyr–Htyr, Owat–Hwat and Oser205–Hser205), sharp distributions with well-defined maxima (values around 1.015 Å) are observed in the ground state, suggesting a definite covalent nature of such bonds and no driving force to react. As a matter of fact, these distances oscillate regularly in time, with average values of around 1.020 Å and a standard deviation of less than 0.030.

Regarding the hydrogen bond (Htyr–Owat, Hwat–Oser205 and Hser205–Oglu222) and the oxygen–oxygen (Otyr–Owat, Owat–Oser205 and Oser205–Oglu222) distances, wider distributions are observed in good agreement with their non-covalent nature, with average values of around 1.580 Å and 2.580 Å, respectively. Distributions involving atoms of the water molecule present smooth curves without well-defined maxima, in accordance with the higher water mobility. Instead, non-bonded distance distributions involving Ser205 show a more peaked shape with defined maxima at around 1.507 Å and 2.522 Å for the Hser205–Oglu222 and Oser205–Oglu222 distances, respectively. This result suggests a crucial role of the protein matrix in modulating the conformation of the atoms in the network. In Fig. 2 we also report the distribution of the chromophore N–C–C–C dihedral angle and of the dihedral angle formed by the oxygen atoms of the hydrogen bond network (Otyr–Owat–Oser205–Oglu222). As we will show in the next paragraph, the dynamics and equilibrium of these parameters are affected by excitation, and this feature helps to explain and clarify the role of the protein environment on photoreactivity. The chromophore dihedral angle shows a very tight distribution centred around –2°, suggesting that the chromophore prefers a planar conformation in its ground state. Concerning the oxygen atoms’ dihedral angle, a spread distribution is observed with values almost focused around 2.5°, even though other populated regions appear in the –20° to –10° and the 20–40° intervals.

### 3.2 GFP excited state AIMD

#### 3.2.1 Trajectory I: the excited state proton shuttle in time and frequency domain

We first discuss results obtained from the S_1_ AIMD trajectory I (TRJI), whose starting configuration is close to the energy minimum in the ground state. Structural parameters mostly affected by the ESPT reaction are analysed in both the time and the frequency domain *via* wavelet power spectra. Simultaneously, we carry out the inspection on the corresponding S_0_ AIMD, performed with the same initial coordinates and momenta. The comparison of the trajectories in S_0_ and S_1_ is very instructive. As a variance of what is observed in the ground state, the rearrangement enforced by the change of the electronic state leads to the ESPT event in 720 fs, with a concerted and almost synchronous mechanism.

**Fig. 3:**
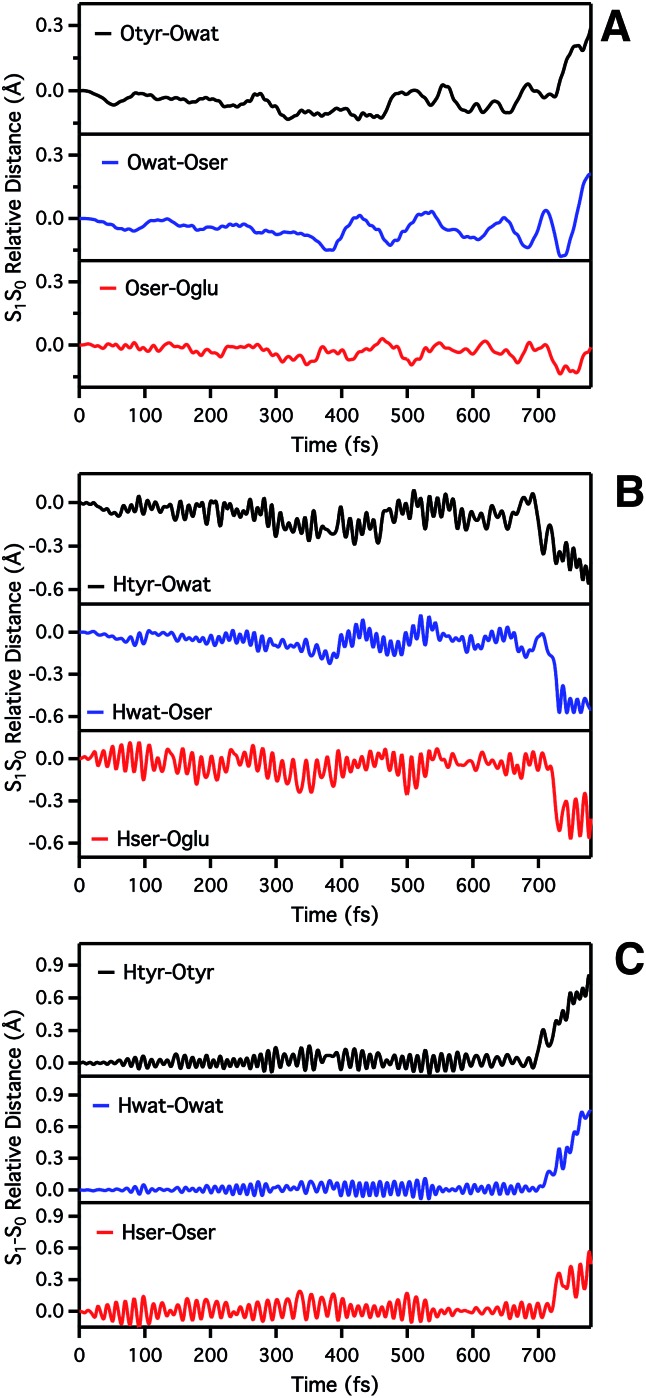
Time evolution of S_1_–S_0_ relative distances (Å) involved in the ESPT. (A) Oxygen–oxygen distances: Otyr–Owat (top), Owat–Oser205 (middle) and Oser–Oglu (bottom); (B) hydrogen–acceptor oxygen distances: Htyr–Owat (top), Hwat–Oser205 (middle) and Hser–Oglu222 (bottom); (C) hydrogen–donor oxygen distances: Otyr–Htyr (top), Owat–Hwat (middle) and Oser205–Hser205 (bottom).

We start analyzing the time evolution of distances between atoms belonging to the hydrogen bond network of the proton shuttle. In Fig. 3 we show the differences between the distance values found in S_1_ and S_0_, respectively.

By inspection of the oxygen–oxygen distance dynamics (Fig. 3a) we observe that in about 15–20 fs the S_0_ and S_1_ curves depart from each other, showing shorter values in the excited state (negative values of S_1_–S_0_ difference). This trend is particularly evident for distances involving the water molecule (Otyr–Owat and Owat–Oser205). These results suggest that the forces acting on the oxygen pairs are different in S_1_ and that the chromophore excitation is able to have an immediate echo on the network, especially on the less rigid and tight part.

In accordance with closer oxygen–oxygen distances, hydrogen–oxygen distance dynamics (Fig. 3b and c) show oscillations mirroring those occurring in S_1_, manifesting a clear departure from the trend in S_0_ in about 100 fs. Overall, the range of values explored in S_1_ is approximately twice as large as in S_0_, eventually leading to the ESPT event in 720 fs.

**Fig. 4:**
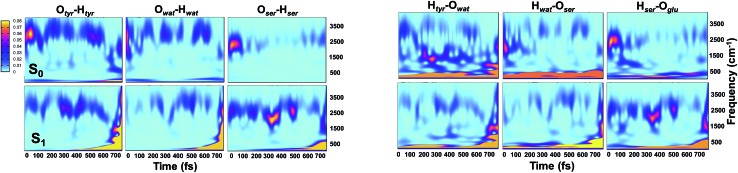
2D wavelet spectra of structural parameters extracted from S_0_ and S_1_ (TRJI) AIMD. (Left panels) Spectra from hydrogen–donor oxygen distances (Otyr–Htyr, Owat–Hwat and Oser205–Hser205). (Right panels) Spectra from hydrogen–acceptor oxygen distances (Htyr66–Owat, Hwat–Oser205 and Hser205–Oglu222). In these spectra, the color represents the magnitude of the wavelet transform (arbitrary units).

Analysis of hydrogen–oxygen distance dynamics in the frequency domain illuminates the forces in play in the two electronic states. We calculated wavelet power spectra of hydrogen–donor oxygen (Otyr–Htyr, Owat–Hwat and Oser205–Hser205) and hydrogen–acceptor oxygen (Htyr–Owat, Hwat–Oser205 and Hser205–Oglu222) distances in both the S_0_ and the S_1_ states. The resulting 2D wavelet maps are reported in the panels in Fig. 4. From inspection of the hydrogen–donor oxygen wavelet maps we observe that the spectra are mainly composed of frequency bands around 3000 cm^–1^, associated to the stretching modes of covalent O–H bonds. The Otyr–Htyr stretching mode appears to be the most energetic, followed by Owat–Hwat and Oser–Hser. Importantly, in the excited state a clear red shift of such bands, starting in the first 100 fs, can be observed. This finding supports the hypothesis of covalent O–H bonds weakened after the excitation, mirroring the wider oscillations of the O–H distances observed in the previous analysis in the time domain. This result is also in agreement with the harmonic frequency analysis performed on the S_0_ and S_1_ energy minima of GFP models (see ESI†). As a matter of fact, we found frequency values for Otyr–Htyr, Owat–Hwat and Oser205–Hser205 stretching modes of 3330, 3099 and 2533 cm^–1^ and 3108, 2859 and 2056 cm^–1^ in S_0_ and in S_1_, respectively. Also, a different temporal evolution of such bands in the two electronic states is clearly observed. In S_1_ the frequency bands show more pronounced oscillations during the time, particularly in proximity to the proton transfer event (700 fs). In particular, the S_1_ Otyr–Htyr spectrum shows an increased red shift after 500 fs. The reaction event in S_1_ is also clearly demonstrated by the appearance of important contributions in the low frequency region at about 700 fs, which are absent in the S_0_ spectra. These indicate the loss of the covalent character in the hydrogen–donor oxygen interactions.

Concerning the hydrogen–acceptor oxygen 2D wavelet maps (right panels of Fig. 4) the bands associated to the O–H stretching modes at about 3000 cm^–1^ are again observed, although they are less intense compared with the ones analysed above. These spectra also show significant differences between S_0_ and S_1_. The S_1_ spectra are indeed affected by the reaction event, showing a clear increase of contributions in both the low and high frequency region close to the reaction time (after 600 fs). Notably, the Htyr–Owat spectrum in the ground state shows contributions at around 1000 and 1500 cm^–1^ which are absent in that of S_1_. We can assign these frequency values to a coupled Otyr–Htyr bending with a water vibration and to the C–O stretching of the tyrosine ring, in agreement with the normal mode analysis. In the excited state, the water-chromophore modes are less coupled. Instead, in the Hser205–Oglu222 S_1_ spectrum a contribution which is absent in S_0_ is clearly observed in the dynamics at around 2000 cm^–1^ and is attributable to the glutamic acid modes.

#### 3.2.2 The photoinduced rearrangement of the chromophore and the chromophore pocket

From the analysis of TRJI in both the time and frequency domain we observe that in S_1_, covalent O–H bonds in the hydrogen bond network weaken while O–O distances shorten; moreover, the evolution of these bonds is affected by the frequency modes of the chromophore, although in a different manner in the two electronic states.

The role of the protein matrix in the GFP ESPT is still under debate. The FSRS results of Mathies and coworkers suggest that the ESPT promoting mode is a photoexcited wagging of the phenolic ring of the chromophore at about 120 cm^–1^. This motion would trigger the approach of the phenolic ring to the hydrogen bonded water molecule, leading eventually to the ESPT.^4^ This mode would also modulate the electronic relaxation in the chromophore skeleton, mainly occurring through out-of-phase bands at 1280 and 1550 cm^–1^, respectively attributed to stretching motions of the phenolic C–O and imidazolinone C–N bonds, whose bond orders are rearranged upon the excitation. In particular, time resolved peaks show a period of 260 fs between maxima. However, this hypothesis of causality between a photoactivated low frequency mode and the ESPT has been questioned in recent works.^26^,^33^

The accuracy of our excited state *ab initio* dynamics and the wavelet approach allows us to give an important contribution to this debate. In the following sections we analyse the chromophore and its pocket rearrangement in both the time and frequency domains, and investigate the connection between the structural reorganization and the promotion of the ESPT.

**Fig. 5:**
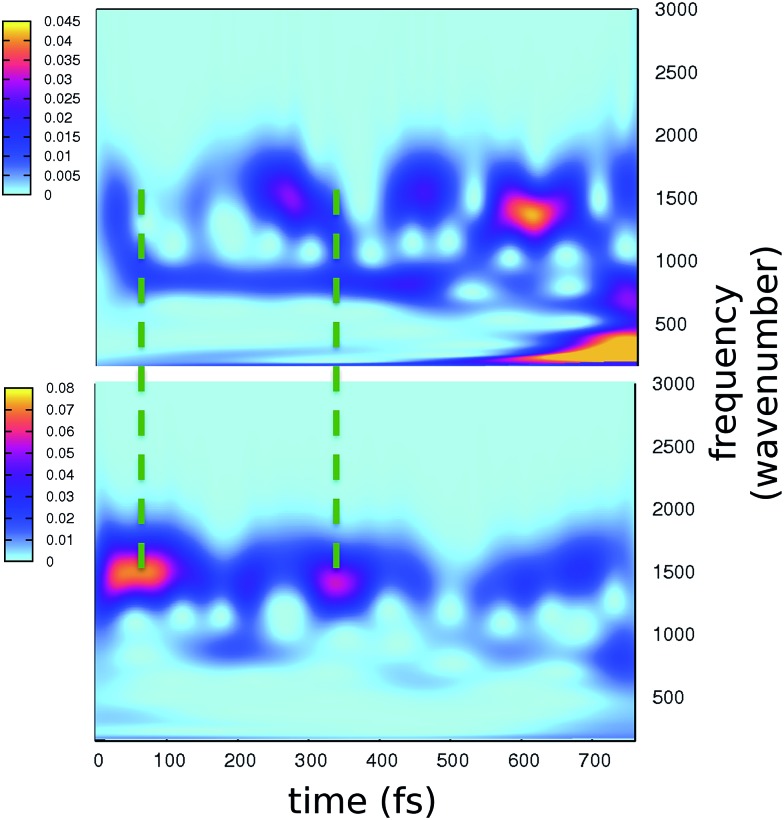
2D wavelet maps of phenolic C–O (top) and imidazolinone C–N (bottom) distances extracted from TRJI. In these spectra, the color represents the magnitude of the wavelet transform (arbitrary units).

The time resolved wavelet power spectra of the phenolic C–O and imidazolinone C–N distances are shown as 2D maps in Fig. 5. We recall that our wavelet spectra represent the vibrational rearrangement following and accompanying the electronic reorganisation of the chromophore, while experimental FSRS delivers Raman activity of modes upon electronic excitation. In spite of this intrinsic difference, the maps in Fig. 5 show an excellent agreement with trends of the FSRS signals.

The main bands in Fig. 5 appear at around 1500 and 1550 cm^–1^ for the C–O and C–N distances, respectively. From our static harmonic frequency analysis on a GFP reduced model and from previous studies,^15^,^63^–^65^ these bands are easily attributed to modes containing C–O and C–N stretching contributions, respectively. In particular, the C–O dynamics are mainly driven by a mode involving a collective bending and deformation motion of the phenolic ring.

Notably, a clear out-of-phase trend of these bands is observed during the time, namely the same behaviour shown by Raman activated C–O and C–N modes. Moreover, the period between the most intense peaks is about 250 fs, again in excellent agreement with FSRS data.

Our results strongly suggest that the time evolution of Raman activated modes from ref. 4 follows the natural route indicated by the electronic relaxation on the single potential energy surface in the excited state, namely what we analyze here, and that the interpretation given by the authors of ref. 4 is largely correct. That means the force relaxation in the chromophore does not occur monotonically, but shows instead a clear oscillatory pattern, which is modulated by the low frequency modes.

On the other hand, due to the full accordance with the experimental trends, we can reasonably assume that the most important features of the experimentally observed rearrangement are captured by our analysis.

**Fig. 6:**
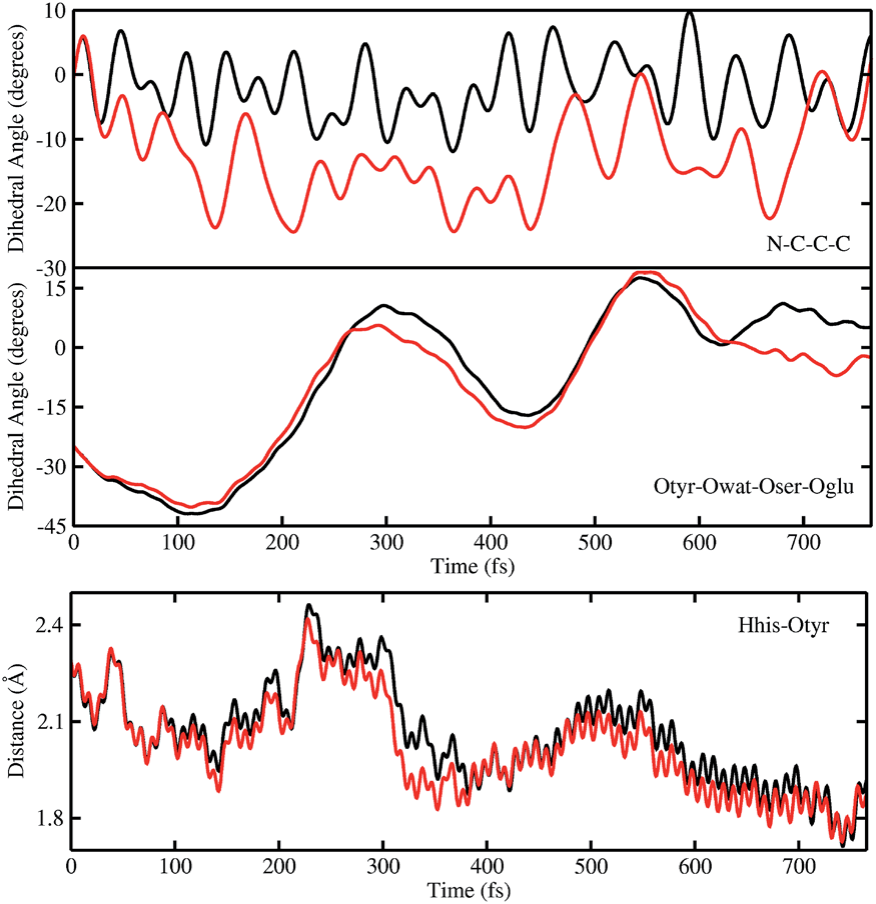
Comparison of distances (Å) and dihedral (degree) angles dynamics obtained from S_0_ (black) and TRJI S_1_ (red) AIMD. The chromophore N–C–C–C dihedral angle (top) and the dihedral angle of oxygen atoms involved in the hydrogen bond network Otyr–Owat–Oser205–Oglu222 dynamics (middle) are shown. Dynamics of the histidine148 proton–OTyr distance are also shown (bottom).

In order to investigate the causality between the rearrangement of the chromophore and the reaction, in Fig. 6 (top and middle panels) we show the temporal evolution in S_0_ and S_1_ of the chromophore N–C–C–C dihedral angle and the dihedral angle formed by the oxygen atoms in the hydrogen bond network (Otyr–Owat–Oser205–Oglu222), representing the relative orientation of the phenolic and imidazolinone rings of the chromophore and the planarity of the hydrogen bond wire, respectively.

Regarding the chromophore N–C–C–C dihedral angle, regular oscillations around the average value of –2 degrees can be observed in the ground state. In S_1_, oscillations became irregular and a wider range of angle values is explored from 5 to –20 degrees. The departure from planarity is particularly evident after about 150–200 fs.

The Otyr–Owat–Oser205–Oglu222 dihedral dynamics shown in Fig. 6 (middle panel) explore values between –40 and 20 degrees in both S_0_ and S_1_. After 600 fs, the oxygen atoms reach a sort of planar conformation in concurrence with the ESPT reaction in S_1_, a variation with what occurs in S_0_. It is noteworthy that this angle oscillates along the whole trajectory according to a period of 250–300 fs, corresponding to a low frequency of about 110–130 cm^–1^. Moreover, differences between the evolution in S_0_ and S_1_ of the two dihedral angles appear to be correlated. This can be observed in the interval between 200 and 450 fs, and after 600 fs, in proximity of the reaction.

**Fig. 7:**
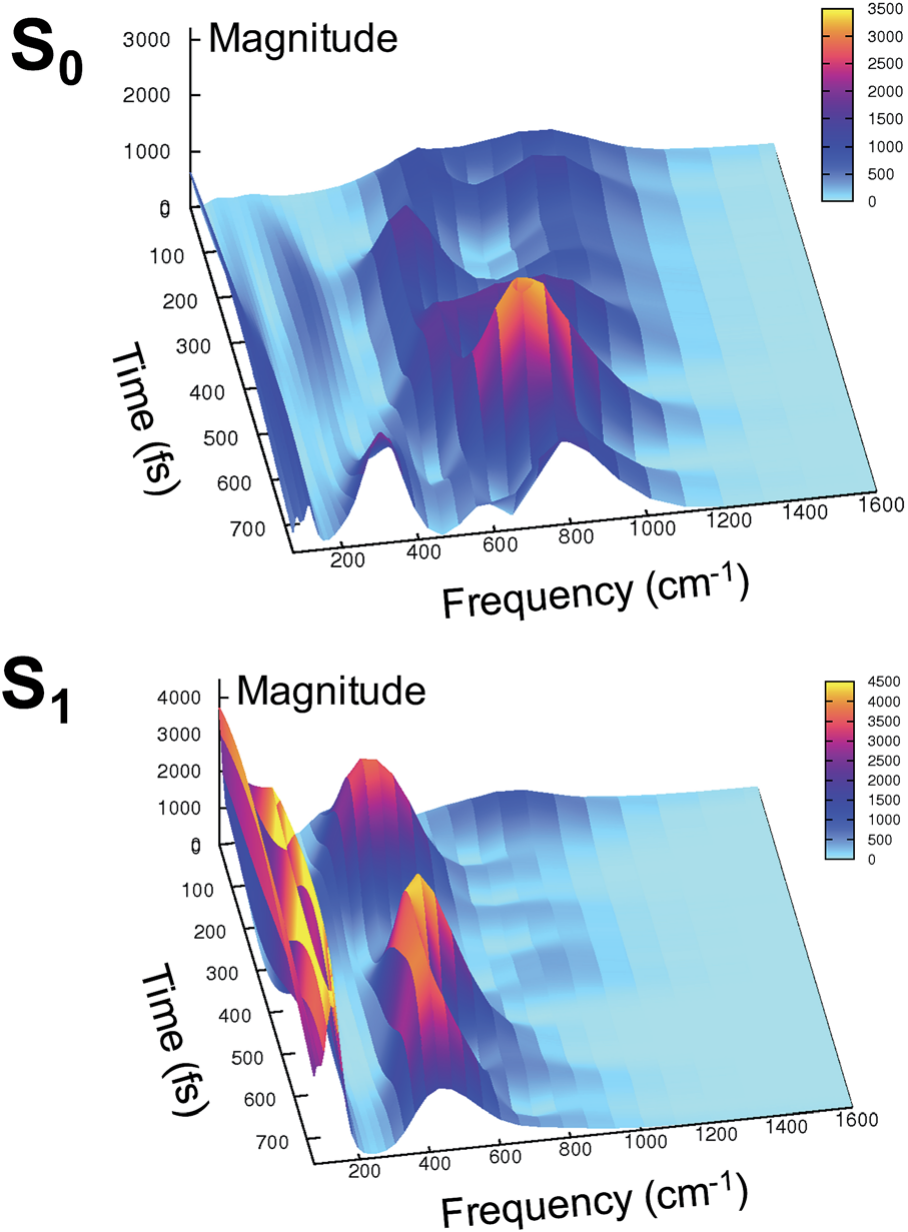
Ground (top) and excited (bottom) state 3D wavelet power spectra of the N–C–C–C chromophore dihedral angle extracted from TRJI. In these spectra, the color represents the magnitude of the wavelet transform (arbitrary units).

At this point it is interesting to discuss the 3D wavelet power spectra of the N–C–C–C dihedral angle obtained in S_0_ and S_1_ and shown in Fig. 7.

In the ground state the main band appears with a frequency around 600 cm^–1^, coupled with close-by minor bands. The spectrum appears significantly different in the excited state, showing one main band centered at around 450 cm^–1^, with no evident coupling with bands that are near during the time. Instead, an intense activation of frequencies below 200 cm^–1^ is observed in the S_1_ spectrum. These results suggest that the relative motion of the two rings is facilitated upon the excitation, and that the relaxation is coupled to the activation of low frequency modes.

**Fig. 8:**
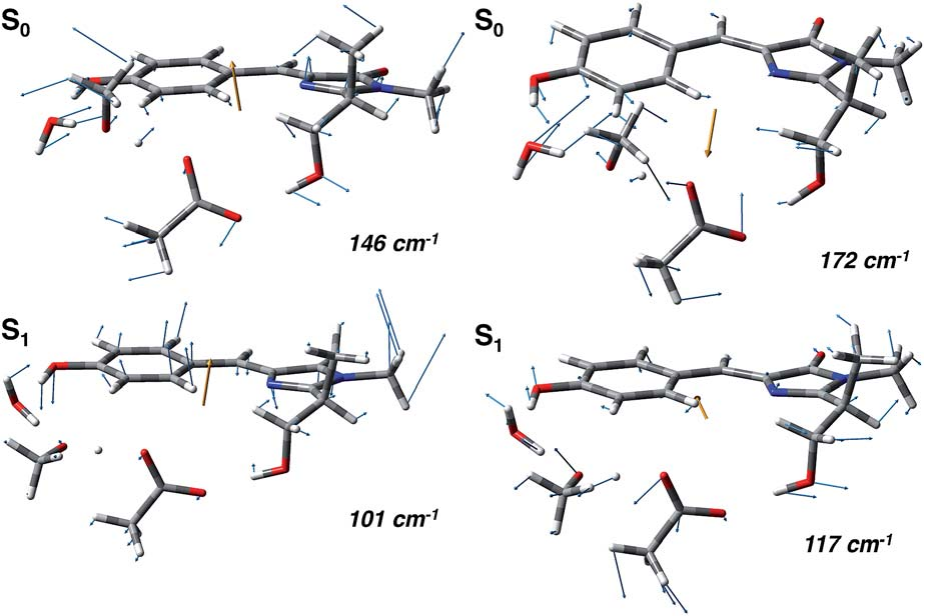
Normal mode analysis of the GFP reduced model involving the chromophore and the residues of the hydrogen bond network performed at the (TD)-B3LYP/6-31+g(d,p) level of theory. (Left panels) Collective mode involving chromophore ring twists in the ground (S_0_, top) and excited state (S_1_, bottom). (Right panels) Collective normal mode mainly involving the phenolic ring and the hydrogen bond network oxygen atoms in the ground (S_0_, top) and excited state (S_1_, bottom).

In agreement with this, from the harmonic analysis in S_0_ and S_1_ on a GFP model, many S_0_ modes involving both the rings of the chromophore (thus affecting the N–C–C–C dihedral) appear to be replaced by modes with single ring contributions in the excited state. Moreover, low frequency modes, involving out of plane motions of the chromophore and oxygen atoms of the hydrogen bond network, are importantly red shifted upon the excitation. A collective mode involving a twist of the chromophore rings is shown in the left panels of Fig. 8 as calculated in the ground (top) and excited (bottom) state. The corresponding frequency shifts from 146 to 101 cm^–1^ when going from the ground to the excited state.

A second mode, involving the out of plane O–H group of the phenolic ring and the oxygen atoms of the hydrogen bond network, is shown in the right panels of Fig. 8. The vibrational frequency shifts from 172 to 117 cm^–1^.

These red shifts are clearly promoted by the softening of the chromophore torsion, and facilitate the tightening of the hydrogen bond network, activating the ESPT reaction coordinate. It is also reasonable that these modes are coupled to the chromophore vibrational relaxing, as can be inferred by their activation in the excited state observed in Fig. 7 and from the oscillatory patterns shown in Fig. 5.

Finally, in Fig. 6 (bottom panel) we report the time evolution of the hydrogen bond distance Hhis148–Otyr involving the phenolic oxygen of the chromophore and the N_δ_H group of His148. His148 has been considered important in modulating the proton shuttle in the ground state,^23^ and also in stabilizing the anionic species I and B.^17^ After 300 fs we can observe a decrease of the hydrogen bond distance in both the S_0_ and the S_1_ dynamics, although in S_1_ the approach of the His148 to the chromophore is more pronounced. Interestingly, this distance is below 2 Å in concurrence with the ESPT event.

In summary, the electronic rearrangement in the chromophore can be accounted for by monitoring the activation of stretching vibrational modes (here, by observing wavelet spectra of C–N and C–O bonds). The chromophore reorganization also implies an important gain of conformational freedom in the chromophore, as witnessed by the lowering of the frequency associated to the N–C–C–C dihedral angle (wavelet spectra in Fig. 7). The vibrational dynamics of the chromophore relaxation are coupled and modulated by low frequency modes, collective in nature and involving both the chromophore and its pocket.

Indeed, our harmonic analysis shows collective modes mainly composed of out of plane motions of the chromophore rings and involving the residues in the network, which in S_1_ are redshifted to values close to 100–120 cm^–1^. The Otyr–Owat–Oser205–Oglu222 dihedral angle, accounting for the relative orientation of the phenolic O–H group with the remaining hydrogen bond wire, evolves with a period corresponding to about 110–130 cm^–1^ and is actually affected by the excitation, assuming a planar arrangement in proximity of the ESPT event. The motions ruling this evolution correspond to the previously presented modes, whose frequencies are slightly redshifted with respect to the dihedral angle oscillating frequencies due to the modelling strategy employed in the normal mode analysis.

Therefore, the ESPT appears to be promoted by the chromophore conformational relaxation, which is concerted and coupled to the relaxation of the hydrogen bond network, which can be accommodated in an arrangement more favourable to the ESPT. As for what constitutes the role of the histidine 148, the observed dynamics suggest that the residue promotes and stabilises the just formed anion.

#### 3.2.3 GFP excited state AIMD trajectories II–IV: general trends in the ESPT mechanism

In this section we discuss the remaining excited state trajectories labelled TRJII, TRJIII and TRJIV. The starting point structures, randomly chosen with their velocities from the S_0_ AIMD dynamics, can be thought of as representative of the Franck–Condon region in the ground state at the instant of photoexcitation.

The ESPT reaction takes place in all the analysed trajectories within one picosecond, namely in 350, 380 and 980 fs in TRJII, TRJIII and TRJIV, respectively.

The observed ESPT kinetics are faster with respect to the experiments. We are exploring the photorelaxation of GFP structures close to the energy minimum, where interactions within the hydrogen bond pocket of the chromophore pocket are optimized, *i.e.* with a propensity to react. This means that our experimental counterpart is the fast part of the real kinetics (few ps). However, our simulations show accelerated reactions, possibly due to the DFT reaction barrier height underestimation and the neglect of polarization effects on the residues of the MM region and close to the chromophore network, such as His148.

A more accurate description of the kinetics would have required the computation of a larger number of excited state trajectories and the use of high level wavefunction methods,^17^,^25^,^26^,^34^–^36^,^39^,^40^ for example by adopting precomputed and high level parameterized potentials, and also including quantum effects.^25^,^26^,^39^,^40^ However, these treatments are unfeasible when including dynamical effects of the chromophore and the entire protein, with no structural restraint in the excited state.

A complete assessment of the GFP ESPT kinetics is beyond the scope of this paper. Indeed, our modelling is specifically designed to give an accurate and effective description of the complex photoinduced vibrational dynamics, and of the ESPT mechanism: the overall timing of the ultrafast relaxation, including that of oscillatory patterns in Fig. 5, is in excellent agreement with experiment. This means that the adopted level of theory is accurate enough to describe the forces and dynamics in play for heavy atoms, and, as a consequence, to describe the overall mechanism leading to the ESPT.

All the trajectories show similar events promoting the ESPT and ESPT mechanism. The ESPT mechanism is concerted and slightly asynchronous in all cases. A tightening of the oxygen–oxygen distances of the hydrogen bond network in proximity to the reaction is always observed (see the figures showing the structural parameter evolution in the ESI†). A common behavior, similar to that previously discussed for TRJI, can be clearly observed in the structural rearrangement of the active site occurring before the reaction event. In all cases the N–C–C–C dihedral angle departs from the initial approximate planarity through ample oscillations, to reach absolute maximum values of 20–30 degrees. During the same time, the dihedral representing the planarity of the oxygen atoms in the network (Otyr–Owat–Oser205–Oglu222) shows a distinct evolution in each trajectory. However, despite the different initial arrangement (values of ≈ –10°, –20° and –10° in TRJII, TRJIII and TRJIV, respectively), it assumes a value close to planarity in proximity of the reaction. Oscillations show periods of 150–220 fs.

The significant role of His148 in the reaction mechanism is confirmed: independent to the initial value (2, 4 and 3.5 Å in TRJII, TRJIII and TRJIV, respectively), a decrease of the Hhis148–Otyr distance is observed about 200 fs before the ESPT event, when it assumes a value of ≈2 Å, as already obtained for TRJI.

In summary, in order for the ESPT to occur the chromophore and its pocket arrangement have to be optimized upon excitation. The loss of the chromophore ring coplanarity (values of the N–C–C–C dihedral far from planarity) in concert with the rearrangement of the hydrogen bond wire have to take place. At the same time, the approach of His148 to the chromophore phenolic ring also supports the reaction.

## 4 Conclusions

In this work we discussed a theoretical-computational investigation of the ESPT reaction in GFP. Our study aimed to investigate the whole protein through accurate *ab initio* molecular dynamics simulations in both the ground and the excited states, and to unveil the driving forces and mechanism of the ESPT reaction through a time-resolved wavelet analysis.

We observed that low frequency vibrational modes are an active part of the ESPT event. Indeed, the ESPT has to be preceded by the activation in the excited state of low frequency vibrational bands modulating the rearrangement of the chromophore N–C–C–C dihedral angle. Simultaneously, the His148 residue approaches the chromophore before the ESPT in all the analysed trajectories, therefore implying that the solvation of the just formed anionic species is important in order to promote the ESPT reaction. The obtained results are in agreement with the hypothesis of a necessary structural reorganization before the reaction, also confirming the capabilities of our approach compared with the results from advanced time resolved spectroscopic techniques.

## 5 Computational details

GFP was modelled starting from the crystallographic structure of wild type GFP from jellyfish *Aequorea Victoria* (PDB code ; 1GFL).^66^ Coordinates were augmented with hydrogen atoms by MolProbity.^67^ Arg and Lis residues were considered positively charged and Asp and Glu negatively charged. We protonated six histidines in both *δ* and *ε* positions, while of the remaining three, two were protonated in *δ* (one of these is his148) and the other one in the *ε* position. All the internal crystallographic water molecules were protonated and retained.

For the ONIOM high level of theory we used B3LYP^68^/6-31+G(d,p) and TD-CAM-B3LYP^69^/6-31+G(d,p) for the ground and the excited states, respectively. The ONIOM low level of theory was provided by AMBER force field,^70^ including the parameters specifically developed for the chromophore by Reuter.^71^ The electronic embedding scheme^53^,^72^ was adopted to account for the interactions between the two layers. The adopted modelling procedure, combined with the starting crystallographic structure, has been previously shown to accurately reproduce the optical properties of GFP.^73^

We collected an AIMD trajectory on the S_0_ PES for 7 ps, employing a time step of 0.5 fs. Excited state trajectories were collected for less then 1 ps with a time step of 0.5 fs. We validated the choice of two different functionals for the ground and excited state AIMD by calculating CAM-B3LYP harmonic frequencies on a reduced GFP model (data not shown). We obtained results very similar to the B3LYP analysis, suggesting that in the ground state the two functionals deliver a consistent description of potential energy surface curvature. All the calculations were performed by the Gaussian suite of programs.^74^

## Conflicts of interest

There are no conflicts of interest to declare.

## Supplementary Material

Supplementary information
