# Supplementary material for: The mechanism of a green fluorescent protein proton shuttle unveiled in the time-resolved frequency domain by excited state ab initio dynamics
Source: Chem Sci. 2018 Jan 2;9(5):1126–35. doi: 10.1039/c7sc02803b (PMC5890789; doi:10.1039/c7sc02803b)
Supplement: Supplementary file 1 [file SC-009-C7SC02803B-s001.pdf]

Electronic Supplementary Material (ESI) for Chemical Science.
This journal is © The Royal Society of Chemistry 2018

Electronic Supplementary Information
The mechanism of Green Fluorescent Protein proton shuttle unveiled in the time-resolved frequency domain by excited state ab-initio dynamics

Greta Donati,[a†] Alessio Petrone,[a]†, Pasquale Caruso[b] and Nadia Rega *[ab]

1 Harmonic frequencies analysis on a GFP model

A GFP model was considered for the harmonic vibrational analysis in both the ground and excited states, including the chromophore and the Ser65 residue, the hydrogen bonded crystallographic water and the residues Ser205 and Glu222. All the calculations were performed at the (TD)-B3LYP/6-31+G(d,p) level of theory.

Displacement vectors for the chromophore twisting mode are shown in Figure 1.

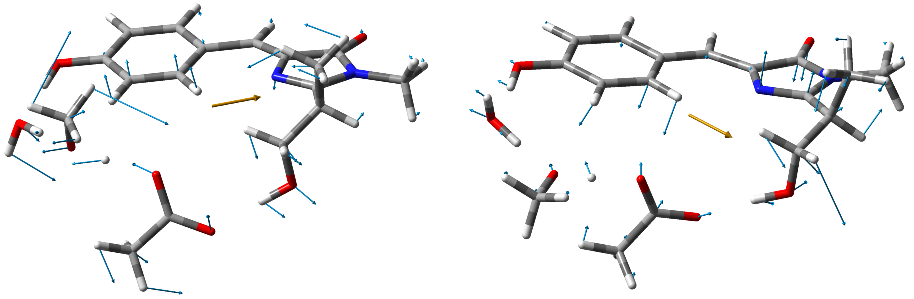

Figure 1 Normal modes analysis of the GFP model involving the chromophore and the residues involved in the hydrogen bond network performed at at (TD)-B3LYP/6-31+g(d,p) level of theory. Chromophore twisting mode in the ground (left) and excited (right) state.

The mode is red-shifted when going from the S_0 to the S_1 state, and also shows a different composition. A wider amplitude of the displacement vectors and the red shift (56.71 cm^{-1} in S_1 vs. 90.80 cm^{-1} in S_0) in the excited state proves a larger conformational freedom of the bridge connecting the two rings, as a consequence of a redistribution of the electronic density upon the excitation on this region.

In Figure 2 the intramolecular O-H stretching modes are shown for both the ground (left panels) and excited (right panels) states.

[0a] *Dipartimento di Scienze Chimiche, Università di Napoli Federico II, Complesso Universitario di M.S. Angelo, via Cintia, I-80126 Napoli, Italy.*
[0†] Current address: *Department of Chemistry, University of Washington, Seattle, WA, 98195*
[0b] *Italian Institute of Technology, IIT@CRIB Center for Advanced Biomaterials for Healthcare, Largo Barsanti e Matteucci, I-80125 Napoli, Italy.*
[0*] *E-mail: nadia.rega@unina.it*

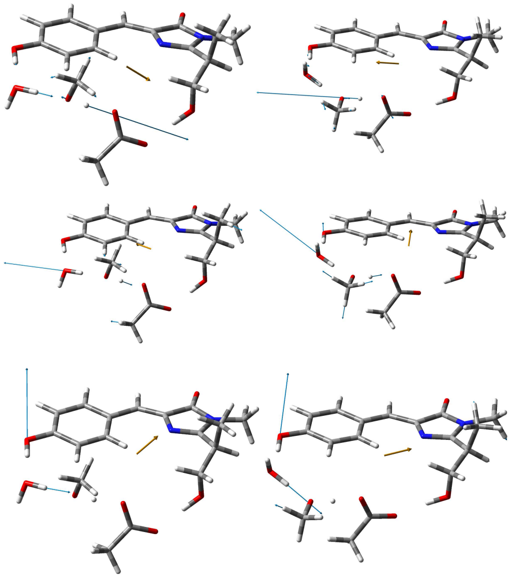

Figure 2 Normal modes analysis of the GFP model involving the chromophore and the residues involved in the hydrogen bond network performed at at B3LYP/6-31+g(d,p) level of theory. Top panel: Ser205 O-H stretching mode in the ground (left) and excited (right) states. Middle panel: Water O-H stretching mode in the ground (left) and excited (right) state. Bottom panel: Phenol ring O-H streching mode in the ground (left) and excited (right) states.

Red shift in the excited state is observed for all the O-H stretching modes. The Oser205-Hser205 is the larger one with a shift value of 477 cm^{-1}. Obviously the frequency values of such modes reflect the different nature of the bonds: 3329.55, 3098.99, 2533.24 cm^{-1} in S_0 and 3108.10, 2859.14, 2056.07 cm^{-1} in S_1 for Otyr-Htyr, Owat-Hwat and Oser205-Hser205, respectively.

The red shift affecting all the analysed modes in the excited state along with some their different composition supports the different dynamics affecting the ground and excited states.

2 GFP excited state trajectories TIJI, TRJII, TRJIII and TRJIV

In Figure 3, is shown the time evolution of structural parameters (Å and degrees) of the GFP chromophore and chromophore pocket extracted from the excited state trajectorys TRJI, compared to corresponding results obtaine from ground state S_0 AIMD.

In Figures 4, 5 and 6 it is shown the time evolution of structural parameters (Å and degrees) of the GFP chromophore and chromophore pocket extracted from the excited state trajectories TRJII, TRJIII and TRJIV, respectively. These trajectories are discussed in the 3.2.3 paragraph of the main paper.

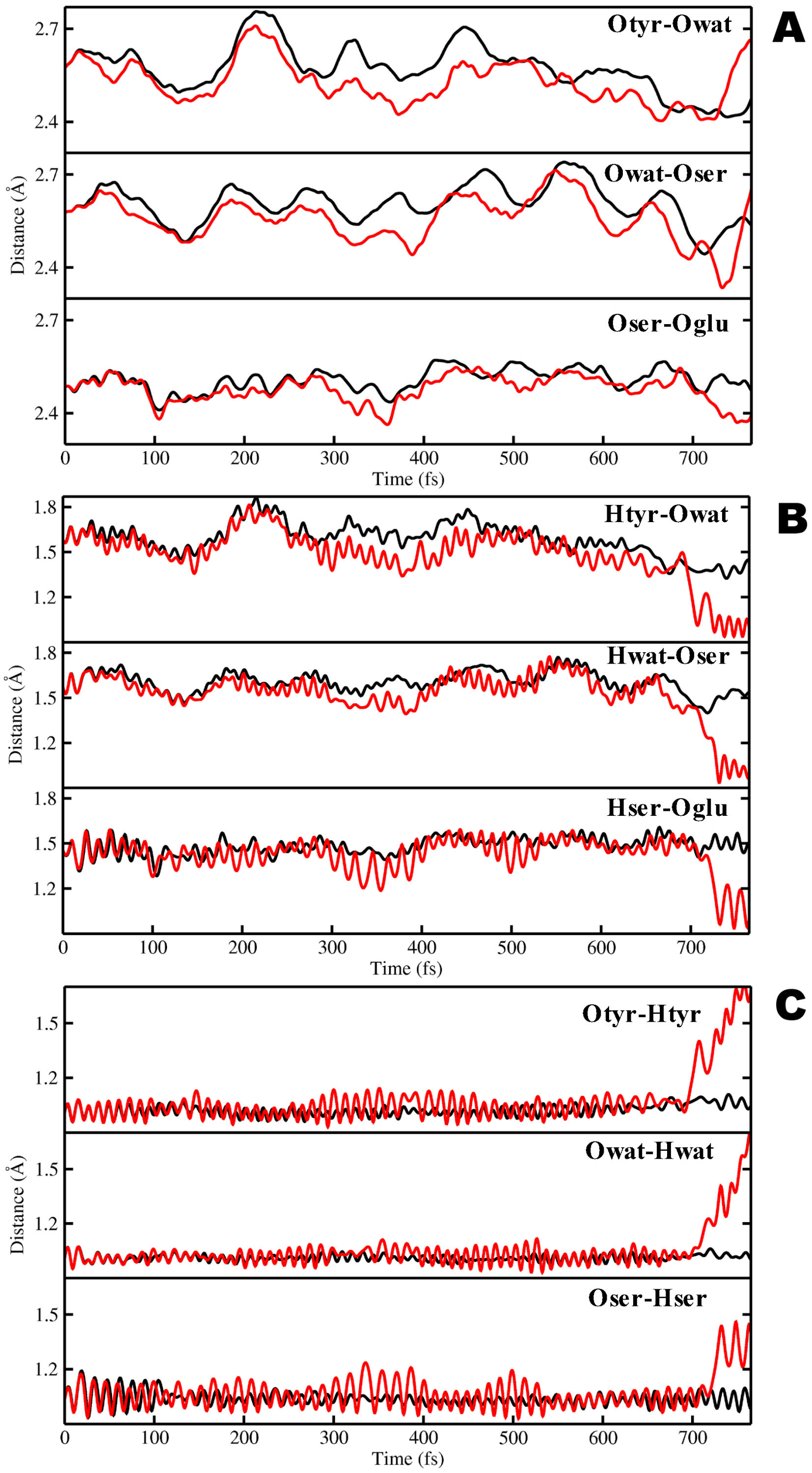

Figure 3 Time evolution of distances (Å) involved in the ESPT. (A) oxygen-oxygen distances: Otyr-Owat (top), Owat-Oser205 (middle), Oser-Oglu(bottom); (B) hydrogen-acceptor oxygen distances: Htyr-Owat (top), Hwat-Oser205 (middle), Hser-Oglu222 (bottom); (C) hydrogen-donor oxygen distances: Otyr-Htyr (top), Owat-Hwat (middle), Oser205-Hser205 (bottom).

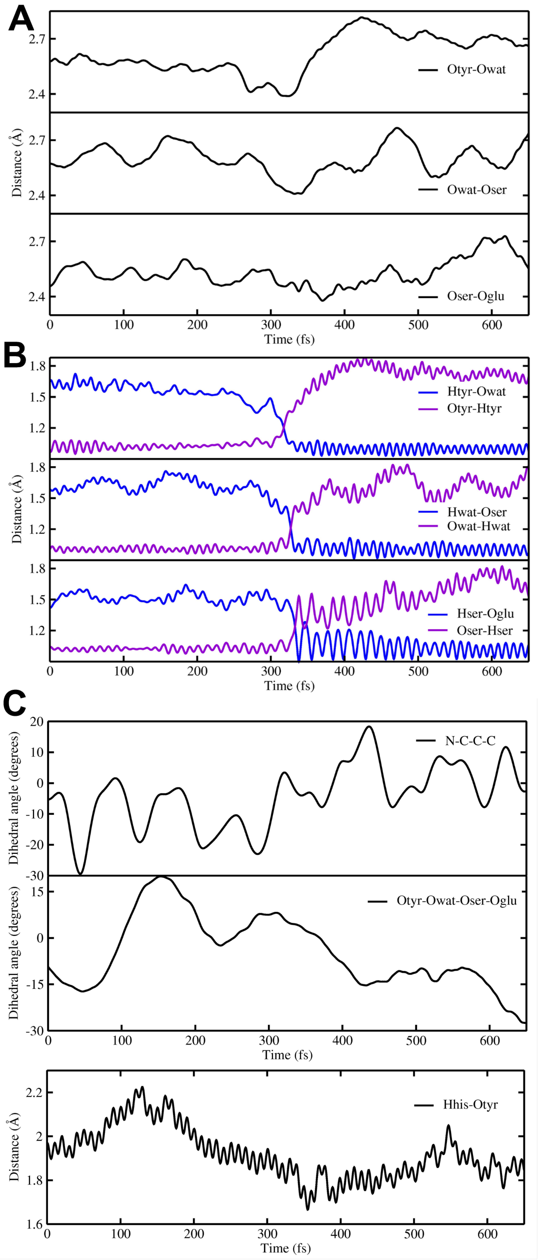

Figure 4 Time evolution of structural parameters (Å and degrees) of the GFP chromophore and chromophore pocket obtained from S_1 AIMD (TRJII). (A) oxygen-oxygen distances: Otyr-Owat (top), Owat-Oser205 (middle), Oser-Oglu(bottom); (B) hydrogen-acceptor oxygen distances (blue lines): Htyr-Owat (top), Hwat-Oser205 (middle), Hser-Oglu (bottom) and hydrogen-donor oxygen distances (violet lines): Otyr-Htyr (top), Owat-Hwat (middle), Oser205-Hser205 (bottom); (C) chromophore N-C-C-C dihedral angle (top), Otyr-Owat-Oser205-Oglu222 dihedral angle (middle), Hhis148-Otyr distance (bottom).

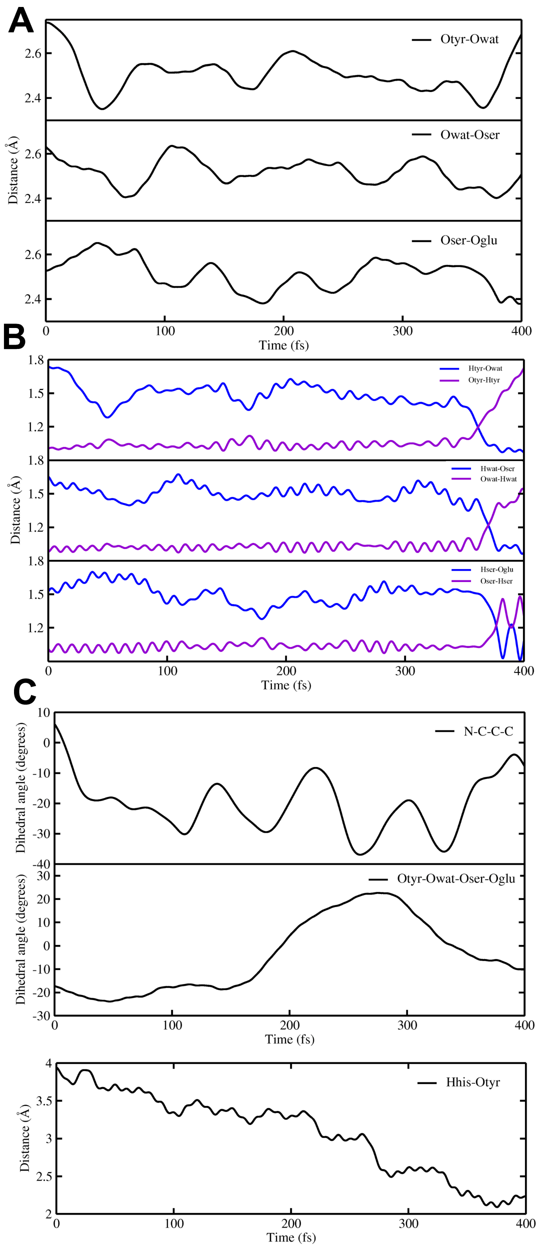

Figure 5 Time evolution of structural parameters (Å and degrees) of the GFP chromophore and chromophore pocket obtained from S_1 AIMD (TRJIII). (A) oxygen-oxygen distances: Otyr-Owat (top), Owat-Oser205 (middle), Oser-Oglu(bottom); (B) hydrogen-acceptor oxygen distances (blue lines): Htyr-Owat (top), Hwat-Oser205 (middle), Hser-Oglu (bottom) and hydrogen-donor oxygen distances (violet lines): Otyr-Htyr (top), Owat-Hwat (middle), Oser205-Hser205 (bottom); (C) chromophore N-C-C-C dihedral angle (top), Otyr-Owat-Oser205-Oglu222 dihedral angle (middle), Hhis148-Otyr distance (bottom).

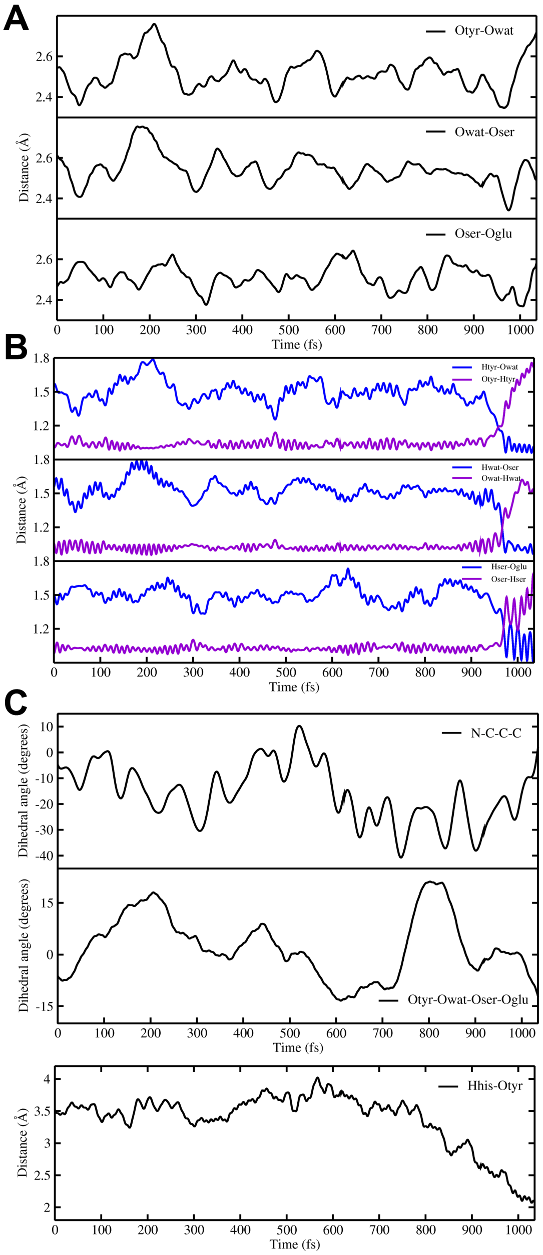

Figure 6 Time evolution of structural parameters (Å and degrees) of the GFP chromophore and chromophore pocket obtained from S_1 AIMD (TRJIV). (A) oxygen-oxygen distances: Otyr-Owat (top), Owat-Oser205 (middle), Oser-Oglu(bottom); (B) hydrogen-acceptor oxygen distances (blue lines): Htyr-Owat (top), Hwat-Oser205 (middle), Hser-Oglu (bottom) and hydrogen-donor oxygen distances (violet lines): Otyr-Htyr (top), Owat-Hwat (middle), Oser205-Hser205 (bottom); (C) chromophore N-C-C-C dihedral angle (top), Otyr-Owat-Oser205-Oglu222 dihedral angle (middle), Hhis148-Otyr distance (bottom).

3 Energetic analysis on the HBDI chromophore

In Figure 7 we show rigid potential energy scans of the anionic form of HBDI chromophore obtained from single point energy calculation for both the ground and excited states at different values of the chromophore dihedral angle N-C-C-C. The chosen range includes values between -30 and 30 degrees in order to cover the wide range of values explored in the excited state protein simulation. These calculations have been performed employing the same potential of the molecular dynamics simulations presented in the main text. Specifically, the analysis was performed on the chromophore in its anionic form in both the ground and the excited states at DFT and TD-DFT level of theory employing the B3LYP and the CAM-B3LYP functionals with 6-31+G(d,p) basis set for S_0 and S_1 calculations, respectively.

Concerning the dihedral angle values from -5 to 5 degrees the same energy values are found for both the electronic states. For values ranging from -14 to -7 and from 7 to 14 the energy difference grows from 0.18 to 0.78 Kcal/mol and from 0.18 to 0.76 Kcal/mol, respectively. This is more emphasized moving from -16 to -30 and from 16 to 30 degrees indeed in the first case the energy difference increases from 1.14 to 3.89 Kcal/mol and in the second case from 1.83 up to 3.87 Kcal/mol.

These results clearly suggest that in the excited state a wider range of dihedral angle values is energetically more accessible to the chromophore. It is also qualitatively expressed by the shapes of the curves. This result is in excellent agreement with the results obtained from our molecular dynamics simulations and vibrational analysis indeed the anionic form is favored by wider N-C-C-C dihedral angles in the excited state. During the simulation as shown in the excited state trajectories, the dihedral angle N-C-C-C explores a range of values larger than in the ground state suggesting that a major conformational freedom is reached by the chromophore after the excitation.

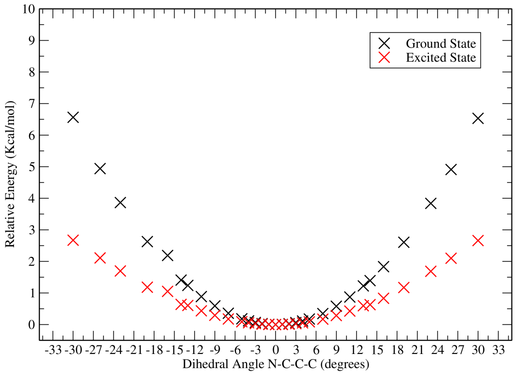

Figure 7 Rigid energy scan of $HBDI^-$ gas phase model along the chromophore N-C-C-C dihedral angle at TD-CAM-B3LYP/6-31+g(d,p) S_1 (red) and B3LYP/6-31+g(d,p) S_0 (black) level of theory. The relative energy corresponding to the reference different minima for both the electronic states are reported in Kcal/mol.
